# Ethics of Giving: The Ratio of Generosity to Income

**Published:** 1915-02-13

**Authors:** 


### ETHICS OF GIVING.

### The Ratio of Generosity to Income.

BY A DRAFTER OF MANY APPEALS.

The announcement recently made, under the heading "Wills Proved," that a testator whose personalty approached £350,000 had bequeathed to his executors "for charitable purposes" "his personal clothing" and nought else, recalls the infrequency with which charitable offerings are made to bear a relation to means. It would seem to most thinkers that a giver who recognises an obligation to charity only to discharge it with flagrant inadequacy is worse than the man who lives wholly oblivious of the claim upon him. At all events, in respect to institutional charity, we may assume as incontrovertible that only in rare instances can a relation be established between gift and means unless it be the relation of diversity. Charitable societies find their most liberal supporters among the moderately well to do, and few workers can fail to perceive how seldom in its treatment of indigent institutions great wealth rises to the occasion. Too often it is concerned only to advise poorer people to subscribe, and one remarkable evidence of philanthropic obliquity is the frequency with which a number of wealthy persons urge the nobodies to support by hard cash undertakings which receive from themselves nothing more than their signatures.

Some experienced persons are led to believe that this is justified in practice because a beggar who is rich starts with an advantage. The average man or woman listens more readily to the pleadings of a rich advocate than a poor one. Those of us who are familiar with Bulwer Lytton's "Money" will remember how, when Alfred Evelyn, the humble private secretary, asked of his rich acquaintances help for his old nurse, they with one accord turned away; but when the same Alfred had succeeded to a big fortune, and by all rules of commonsense, and even common propriety, ought to have provided his own benefactions, his friends fell over one another in their eagerness to give assistance. This picture is as suggestive and as true to human nature as the kindred reflection of Sir John in the same masterpiece, which, paraphrased, runs that if we would that people should believe us rich we must first persuade them that we are stingy.

Even posthumous benevolence is uncultivated among the ultra-rich, and people who have signed appeals in the newspapers, sold wares at bazaars, or in some other way have held out their hands to their friends and the public, and so have achieved a philanthropic reputation, will leave behind them vast fortunes uncharged with so much as a nominal tribute to any one of the causes they laboriously advocated.

Some time ago, when statistics were prepared of bequests to hospitals published in the newspaper lists of wills proved, it was found that of a total personalty of twenty-eight millions of pounds dealt with in a particular year, estates represented by twenty-one millions were disposed of without any offering to charity, and close study of the figures seemed to show that, notwithstanding a few notable exceptions, charity is apt to languish as we mount into great riches, and to disappear altogether, like a frail flora, when the highest altitudes are reached. One testator with £917,000 to bequeath left £1,200 to sundry hospitals, another with £373,000 left £200, and a third £100 out of £694,000, while the four actual millionaires included in the year's record devoted not a penny of their vast wealth to the relief of suffering. If we remember that in some instances the value of the testator's real estate exceeded the personalty, the significance of the figures becomes still more surprising.

One of the difficulties encountered by secretaries and managers is the inability of many people to see in hospitals anything more than places where benefits are dispensed, and to be made use of in order that protégés may be provided for at someone else's expense. How the institutions are maintained is not their concern, and they refuse to consider the question. A simple syllogism suffices. The hospital is there, a hospital's function is to dispense aid; their nominee needs aid, and that is enough. But besides the above there are other explanations of the indisposition of wealth to look upon poverty with unalloyed sympathy. Certain writers have advanced the opinion that poverty may be a crime to be punished rather than a misfortune to be pitied, and that view is not seldom adopted by those who have never felt the want of anything money will procure. They resent the intrusion of unpleasant and unwelcome facts upon a prospect they prefer to be shadowless, and, incredible as it may appear, not a few of them show jealousy of the poor when the golden master-key which is supposed to fit every lock fails.

Happily, examples to the contrary are not wanting. Some poor people who have benefited directly by hospital ministrations will exhibit their gratitude by gifts they can ill afford to make, and the list of subscribers to any given hospital must contain the names of not a few whose contributions have been made with difficulty. This attainment is not limited to the actually poor. Some, who are rich, at any rate relatively, interpret their obligations generously enough to bring themselves well within the category, and perhaps the greatest of all philanthropic achievements is to be munificent though wealthy.

In not a few cases the bond of sympathy is fellow-suffering, and many a hospital has learned how powerful that is. But the complexity of human instincts does not permit of a too general application of this reflection. A man very highly placed, and accustomed to practise a philanthropy both liberal and discriminating, was repelled by the fact that his own child had fallen a victim to one of a group of maladies specially dealt with by a hospital which sought his assistance! Another wealthy and generous helper of many kindred organisations regarded its title as a horrible reminder of his own sufferings, and turned from its work with impatience.

But the fact is that motives of pure reason to explain voluntary action are usually difficult of discovery, and not in charitable spheres only. The application of benevolence, notwithstanding the efforts of disciplinarians and organisers, will never rank among the exact sciences. It is among the trials of poor men, generous of soul, that they cannot satisfy the cravings of their benevolent instincts. But they possess an ineffable advantage over richer donors; they may attain to a self-sacrifice in giving which seasons and glorifies the gift, and is impossible to those who merely transfer to charitable uses a portion of scarcely realised superfluity.

